# Molecular Identification of Two DNA Methyltransferase Genes and Their Functional Characterization in the Anti-Bacterial Immunity of *Antheraea pernyi*

**DOI:** 10.3389/fimmu.2022.855888

**Published:** 2022-05-16

**Authors:** Saima Kausar, Muhammad Nadeem Abbas, Isma Gul, Ruochen Liu, Qianqian Li, Erhu Zhao, Muhan Lv, Hongjuan Cui

**Affiliations:** ^1^ State Key Laboratory of Silkworm Genome Biology, Key Laboratory of Sericultural Biology and Genetic Breeding, Ministry of Agriculture, Southwest University, Chongqing, China; ^2^ Cancer Center, Medical Research Institute, Southwest University, Chongqing, China; ^3^ Department of Psychology, The Second Affiliated Hospital of Chongqing Medical University, Chongqing, China; ^4^ Department of Gastroenterology, The Affliated Hospital of Southwest Medical University, Luzhao, China

**Keywords:** silkworm, epigenetics, DNA methylation, pathogens, antimicrobial peptides

## Abstract

Under different physiological conditions, such as microbial infection, epigenetic mechanisms regulate genes at the transcription level in living organisms. DNA methylation is a type of epigenetic mechanism in which DNA methyltransferases modify the expression of target genes. Here, we identified a full-length sequence of DNMT-1 and DNMT-2 from the Chinese oak silkworm, *A. pernyi*, which was highly similar to the homologous sequences of *Bombyx mori*. ApDNMT-1 and ApDNMT-2 have unique domain architectures of insect DNMTs, highlighting their conserved functions in *A. pernyi*. *ApDNMT-1* and *ApDNMT-2* were found to be widely expressed in various tissues, with the highest levels of expression in hemocytes, the ovary, testis, and fat bodies. To understand the biological role of these genes in microbial resistance, we challenged the fifth instar larvae of *A. pernyi* by administrating Gram-positive and Gram-negative bacteria and fungi. The results revealed that transcript levels of *ApDNMT-1* and *ApDNMT-2* were increased compared to the control group. The inhibition of these genes by a DNMTs inhibitor [5-azacytidine (5-AZA)] significantly reduced bacterial replication and larvae mortality. In addition, 5-AZA treatment modified the expression patterns of antimicrobial peptides (AMPs) in the *A. pernyi* larvae. Our results suggest that *ApDNMT-1* and *ApDNMT-2* seem to have a crucial role in innate immunity, mediating antimicrobial peptide responses against bacterial infection in *A. pernyi*.

## Introduction

The Chinese oak silkworm, *A. pernyi*, is broadly cultured for silk production in Asian countries, such as China, Korea, and India (1). This wild silk moth is also used as a model insect to investigate immunological response, development, metabolism regulation, and breeding technique. *A. pernyi*, like other insects, has strong and efficient innate immunity, including cellular and humoral components. Encapsulation, nodule formation, and phagocytosis are examples of cellular immune responses (2, 3). The humoral immune response includes the production of AMPs following a microbial infection (2, 4). In *A. pernyi* and other insects, complex sets of genes involved in the coordination of the innate immune system are related to the immune pathways such as Toll, Imd, and NF‐κB pathways (5). After pathogen detection, the activated cell surface receptors induce the downstream signaling cascade inside cells, stimulating the targeted immune response. Toll and IMD pathways produce AMPs in response to bacterial and fungal infection (5, 6).

DNA methylation is one of the critical epigenetic modifications that regulate gene transcription in eukaryotes and control various biological processes. DNA methyltransferases are responsible for DNA methylation (7, 8). Compared to mammals, studies on DNMTs and DNA methylation in insects remain relatively poor. However, some progress has been made in insects in recent years due to the development of innovative DNA methylation research methods. DNA methylation has been found in the genomes of insects such as silkworm, *Nasonia Vitripennis, Tribolium castaneum*, and *Drosophila melanogaster*, according to whole-genome bisulfite sequencing studies (9–12). However, the maintenance and regulatory mechanisms of DNA methylation in insects may vary significantly from those in other organisms (13–15). Insects have been shown to have different combinations of DNMT gene sets. The *Apis mellifera* genome contains DNMT-1, DNMT-2, and DNMT-3 genes (16). However, in silkworms and *Tribolium castaneum*, only two DNMT genes (DNMT-1 and DNMT-2) have been identified (16). In the living organisms lacking DNMT-3, including silkworms and beetles, DNMT-1 appears to be a *de novo* and maintenance DNMT. DNA methylation regulates various physiological processes in insects, such as immunity, memory, aging evolution, and others (17–21).

Insect immunological responses have not been extensively explored in terms of DNA methylation’s biological roles. It has been shown that *Aedes aegypti* genome wide-patterns of DNA methylation are disrupted by the infection by Wolbachia (22). Cytoplasmic polyhedrosis virus infection caused the differential expression of genes and affected the DNA methylation status in the fat bodies and midguts of silkworms (23). Subsequently, Wu et al. (15) observed the differential expression of DNMT genes in the *Galleria mellonella* larvae infected with three different strains of *Metarhizium anisopliae*, suggesting that DNA methylation in insects is likely involved in the immune response against microbial pathogens (15). In the present study, we identified the DNA methylation tool kit in the Chinese oak silkworm *A. pernyi*. Then we analyzed their spatial and temporal expression patterns in different tissues of *A. pernyi* larvae. Next, we treated *A. pernyi* larvae with 5-AZA, a DNA methylation inhibitor, and analyzed the effects of DNA methylation inhibition on the insect’s immune responses.

## Materials and methods

### Culture of *A. pernyi* Larvae

The model insect, *A. pernyi* larvae, was maintained at room temperature and fed on fresh oak leaves as described in previous reports (2, 4). Healthy fifth instar larvae were used in all of the following experiments.

### Gene Identification and Bioinformatics Analysis

To identify ApDNMT-1 and ApDNMT-2 sequences, we considered the genomic library of *A. pernyi* constructed in our laboratory. The retrieved sequence was further analyzed by the NCBI blastn tool for confirmation. Then the fragments of these genes were amplified by polymerase chain reaction using specific primers (**Table 1**). The ORF sequences of *ApDNMT-1 and ApDNMT-2* were mapped into their respective sequences. The domain architecture was determined by using the blastp program. Multiple sequence alignments were performed with representative DNMT-1 and DNMT-2 sequences from other insect species retrieved from the GenBank database using Clustal software. The three-dimensional protein structure was analyzed by the SWISS-MODEL (http://Swiss-model.expasy.org). Phylogenetic analysis was carried out using Mega 6 software with 1000 bootstraps using the neighbor-joining method based on amino acid sequences.

**Table 1:** Primers used in this study.

Primer Name	Fwd./Rev.	Purpose	Sequence (5′-3′)	Reference
ApDNMT-1	Fwd.1	Amplification	AAATATTGTTGTTTTTTTAC	This study
	Rev. 1		AAATTGTGCATGCTTAT	
ApDNMT-1	Fwd. 2		GTAGTTGTTGAATACTTGGAGG	
	Fwd. 2		GTCTATGTTGGAACCACGT	
ApDNMT-1	Fwd. 3		GGCAAGACTACGATTATA	
	Fwd. 3		CATGATTAACGGTTTTCC	
ApDNMT-1	Fwd. 4		TAGGAGCGGCTTTGGGTAGAG	
	Fwd. 4		GTACCACAGACGAGAAGAAGACTT	
ApDNMT-2	Fwd.		GGAGAAAAAAAAAAGTCG	
	Fwd.		GTACTACATTTCTCAAACC	
ApDNMT-1	Fwd.	qRT-PCR	ACGACTACGCCGTTGGTAAG	
	Fwd.		TCATCAGCTTGTTCCACAGC	
ApDNMT-2	Fwd.		TGTGCATGGAATGAATCAGG	
	Fwd.		CGGATCATTTTCATCGAGGT	
ApCecropin	Fwd.		TCGTACTTAAGTCGCGTCCT	(2)
	Rev.		GCTACGTTGGTTTCCCGTTT	
ApGloverin-like	Fwd.		CTGGAAGGACAAGCTTACGG	(2)
	Rev.		GATCCTCCAGCTGAGAGACG	
ApLebocin	Fwd.		CTTCGCGATGATGATGCTCC	This study
	Rev.		TACGGGAAGCTGCAGATGAA	
ApAttacin	Fwd.		TGCTCGTAGAAGAGCCTGGT	(2)
	Rev.		GCTCCCAGCTTCATTCTGTC	
ApRelish	Fwd.		TGTGGTGACAATGGCAACTT	This study
	Rev.		GGTGCTCATCGGGATTCTTA	
ApTLR-like	Fwd.		CCTTACATCGGGCATGTTCT	This study
	Rev.		GGAATGAAGGCGTAATCGAA	
ApDorsal	Fwd.		TGAGCATGTCTCCCACTTTG	This study
	Rev.		GTGGACTTGTTGCATGATGG	
Ap18S RNA	Fwd.		CGATCCGCCGACGTTACTACA	(2)
	Rev.		GTCCGGGCCTGGTGAGATTT	
16s rRNA	Fwd.		AGAGTTTGATCMTGGCTCAG	(24)
	Rev.		TACGGYTACCTTGTTACGACTT	

### Tissue Distribution Pattern Analysis of DNMT Genes

To analyze tissue distribution patterns of DNMT genes, hemocytes, fat body, midgut, silk gland, testis, ovary, Malpighian tubules, and integument tissues were collected from the healthy *A. pernyi* larvae. Total RNA from each sample was extracted using TRIzol reagent (Invitrogen) according to the suppliers’ instructions, and 2 μg of total RNA was used to prepare cDNA. Oligonucleotide primers specific for the *ApDNMT-1 and ApDNMT-2* sequences and the 18S rRNA sequence for internal control were designed using the Primer 3.0 online primer designing tool (http://bioinfo.ut.ee/primer3-0.4.0/primer3/) (**Table 1**). We performed PCR and analyzed amplicons on the agarose gel before carrying out qRT-PCR to ensure the integrity and stability of 18S rRNA in the cDNA of all samples used in this study. The quantitative real-time polymerase chain reaction assay was performed with specific primers and synthesized cDNAs, and each reaction mixture contained 20 μL with 10 μL of SsoFast EvaGreen SuperMix (Bio-Rad, Hercules, California, USA), 1 μL of forward primer (200 mM), 1 μL of reverse primer (200 mM), 1 μL of diluted cDNA, and 7 μL of sterile water. The thermal cycling conditions were 95°C for 30 s, followed by 40 cycles of 95°C for 5 s and 60°C for 34 s. Amplification was monitored on the iCycleriQ TM RT-PCR Detection System (Bio-Rad, Hercules, California, USA). The specificity of the SYBR-Green PCR signal was further confirmed by melting curve analysis. The experiments were repeated three times as independent biological replicates. The mRNA expression was quantified using the 2^-ΔΔC^ *T*method (25).

### Microbial Injection and Expression Analysis

For obtaining induction profiles of DNMT genes, fifth instar larvae of *A. pernyi* on the third day were divided into four groups with 54 larvae in each group. The *A. pernyi* larvae were injected with 5 μL each of *E. coli* (DH5α, 1 × 10^6^ cells), *B. cereus* (1 × 10^6^ cells), or *B. bassiana* (1 × 10^6^ spores). The larvae injected with PBS were used as a control group. These microbial organisms were injected into the larval abdominal area using microliter syringes (Gaoge, Beijing, China), and injection sites were sealed with vaseline immediately after injection. The larvae were dissected, and immune tissues, including the fat bodies and hemolymph, were sampled at different time intervals (1.5, 3, 6, 12, 24, and 48 h) after injection. Three larvae were collected as one sample, and the biological sampling protocol was repeated three times. The transcript expression patterns of *ApDNMT-1* and *ApDNMT-2* genes were recorded using a qRT-PCR assay as described in the above section.

### 5-Aza-2-Deoxycytidine (5-AZA) Administration

To determine the effect of 5-aza-2-deoxycytidine on the DNMT genes and, subsequently, on the immune responses against bacterial pathogens. 5-AZA can prevent *de novo* and maintenance of DNA methylation by binding covalently to DNMT genes (26). We purchased 5-AZA and diluted it in distilled water with 40 mM/1 μL. The fifth instar larvae of *A. pernyi* were injected with 1 μL 5-AZA into the abdominal area of the larvae, and the second injection was performed at 24 h intervals. Then we analyzed, the expression patterns of both the *ApDNMT-1* and *ApDNMT-2* genes using qRT-PCR. Bacterial pathogens (*B. cereus* or *E. coli*) were injected 24 h after the second 5-AZA injection. The larvae were used as control groups without any treatment, and the larvae were injected with distilled water.

### Bacterial Load Estimation and Survival Analysis

To estimate the bacterial load, we first analyzed the bacterial clearance ability of larvae injected with 5-AZA and bacterial pathogens. After 6 h of bacterial injection, *A. pernyi* larvae legs were punctured to collect hemolymph from the 5-AZA treated group and control group (distilled water treated and untreated) and then immediately diluted. Fifty μL of each dilution was plated onto agar plates, incubated overnight at 37°C, and the colony-forming units were counted.

Furthermore, total genomic DNA was extracted from the hemolymph samples of treated (bacterial-injected and 5-AZA treated) and control *A. pernyi* larvae. The concentration of the extracted DNA was determined by a spectrophotometer, and 500 ng of total genomic DNA was used for each qRT-PCR reaction, and the analysis was performed as described in section 2.3. Specific primers for bacterial 16s rRNA were used in qRT-PCR, and the *A. pernyi* 18s rRNA gene was used as an internal control for normalization (24, 27). To better understand the insect-pathogen interaction, the mortality of *A. pernyi* was also analyzed using Graphpad Prism 6.0. Kaplan-Meier survival analysis.

### Analysis of AMPs and Their Associated Pathways

*A. pernyi* larvae were treated with 5-AZA- bacteria (*B. cereus or E. coli*) or only bacteria or untreated larvae to determine the response of AMPs and their associated pathways. The larvae were dissected, and the fat bodies were used to isolate total RNA, which was then reverse transcribed to prepare cDNA. A quantitative RT-PCR assay was carried out (described in section 2.3) using AMPs (gloverin-like, cecropin, attacin, and lebocin) and their associated pathway genes by specific primers (**Table 1**).

### Statistical Analysis

In this study, all experiments were executed in triplicate and the obtained data represented the means ± S.E. The one-way analysis of variance (ANOVA) and Tukey’s multiple range tests were used to evaluate the difference between groups.

## Results

### Bioinformatic Analysis of DNMTs of *A. pernyi*

**Figure 1:**
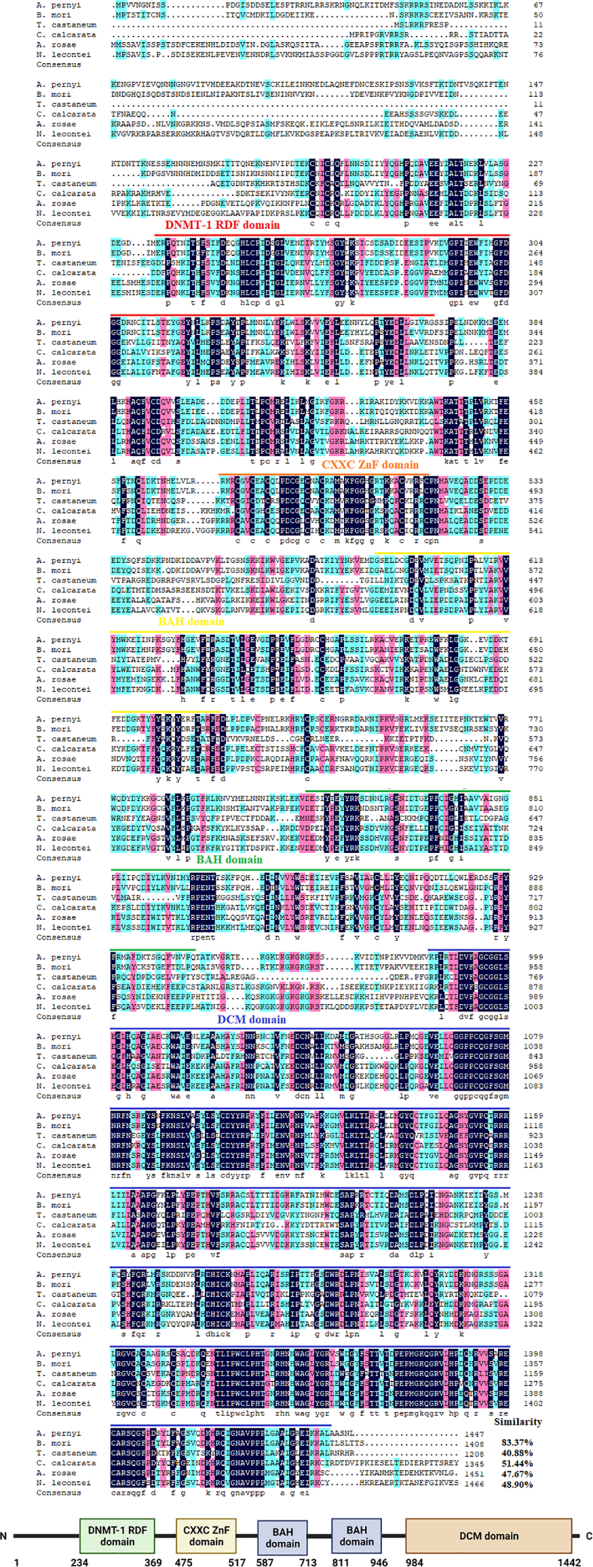
Alignment of the *ApDNMT-1* protein with homologous proteins. The *ApDNMT-1* deduced amino acid sequence was aligned with other DNMT-1 protein sequences. The sequences for alignment analysis are the following: accession no. NP_001036980.1 (*Bombyx mori*), accession no. ASA69505.1 (*Tribolium castaneum*), accession no. XP_026671099.1 (*Ceratina calcarata*), accession no. XP_012254091.1(*Athalia rosae*), and accession no. XP_015517160.1 (*Neodiprion lecontei*). Identified domains were labeled as DNMT1-RDF, DNA methyltransferase replication foci domain which is indicated by a red line; ZnF, Zinc finger domain is indicated by the orange line; BAH, Bromo adjacent homology domain is shown in yellow line; DCM, DNA-cytosine methyltransferases domain is shown in green line Cys-rich, cysteine-rich ADD domain is presented in the blue line.

**Figure 2:**
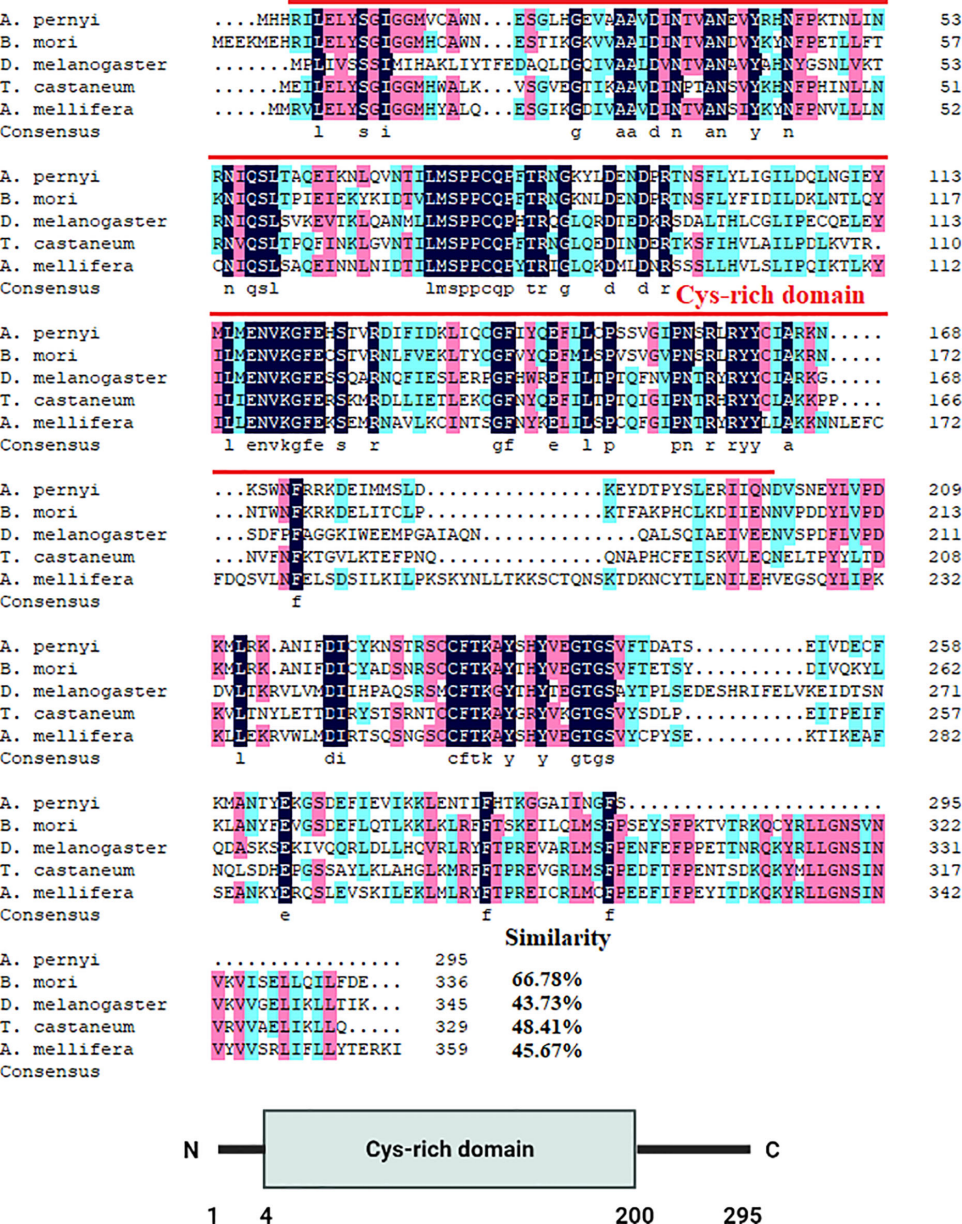
Alignment of the ApDNMT-2 protein with homologous proteins. The ApDNMT-2 deduced amino acid sequence was aligned with other DNMT-2 protein sequences. The sequences for alignment analysis are the following: accession no. NP_001036934.1 (*Bombyx mori*), accession no. AAF03835.1 (*Drosophila melanogaster*), accession no. (*Tribolium castaneum*), accession no. XP_006563008.1 (*Apis mellifera*). The putative DNMT2 cyt_C5_DNA methylase superfamily domain is indicated by red line.

**Figure 3:**
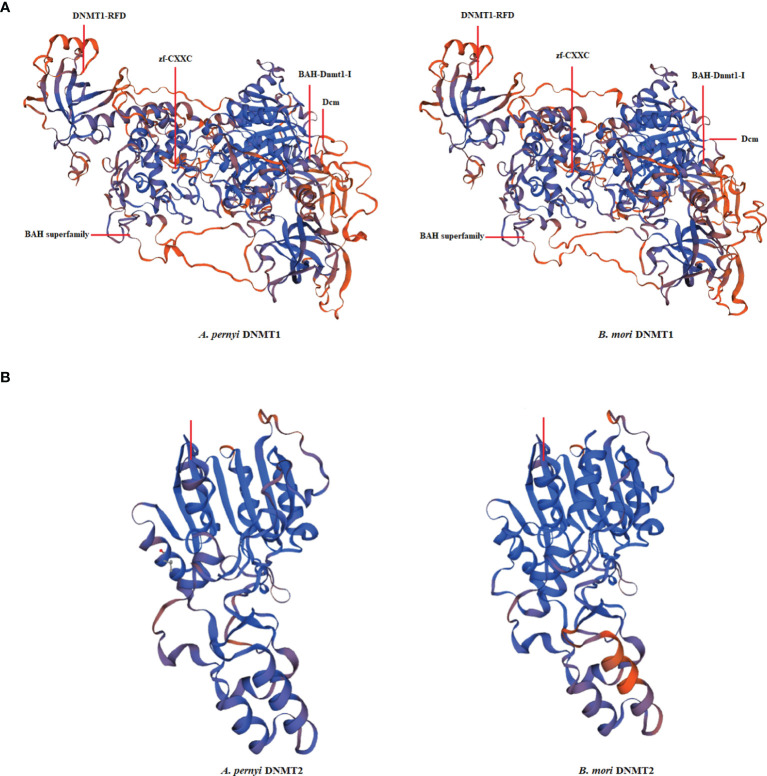
The tertiary structure of *A. pernyi* DNMT-1 **(A)** and DNMT-2 **(B)** proteins in comparison with *Bombyx mori* DNMT-1 and DNMT-2 proteins.

**Figure 4:**
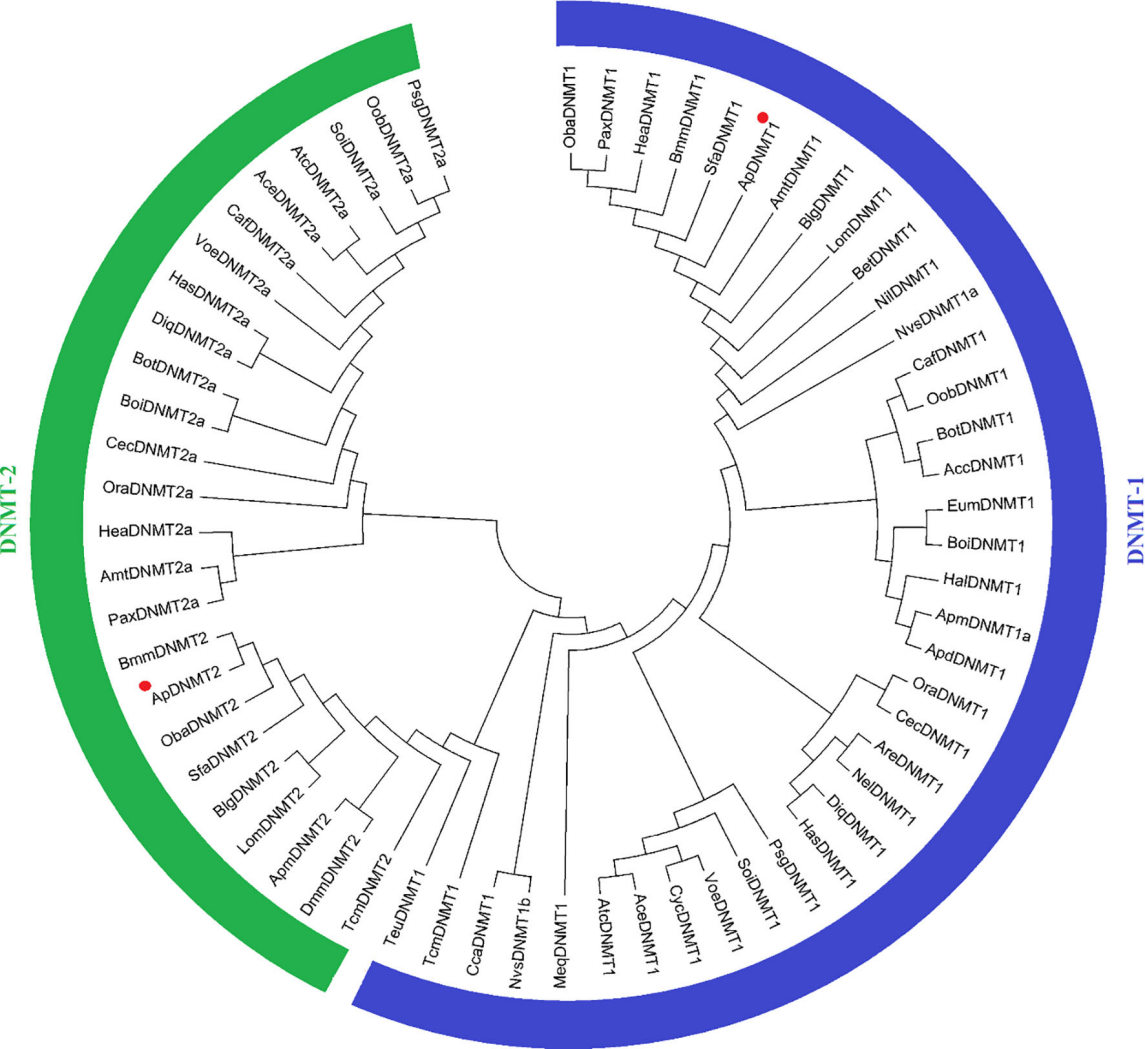
Phylogenetic analysis of *A. pernyi* ApDNMT-1 and ApDNMT-2 proteins with other DNMT-1 and DNMT-2 proteins from vertebrate and invertebrate species. The deduced amino acid sequences were aligned; later a phylogenetic tree was constructed using the neighbor-joining method.

The cDNA sequences of ApDNMT-1 and ApDNMT-2 were identified by using a genomic library of the Chinese oak silkworm *A. pernyi*, which was constructed in our laboratory. The full-length sequence of ApDNMT-1 comprised a total length of 5508 base pairs. The open reading frame (ORF) is 4344 bp and contains a 209 bp 5′ untranslated region (5′ UTR) and a 955 bp 3′ UTR (**Supplementary Figure S1**). The deduced ApDNMT-1 protein is comprised of 1447 amino acids with an approximate molecular weight of 163.182 kDa and isoelectric point of 6.53, respectively. The NCBI Conserved Domain Database was used to determine conserved domains of ApDNMT-1, which showed that this gene has four conserved domains: a Zinc finger domain, a Bromo adjacent homology domain, a DNA-cytosine methyltransferase domain, and a cysteine-rich ADD domain (**Supplementary Figure S1**; **Figures 1**, **3**). Further, it was found that ApDNMT-1 has a comparable structure and domain architecture similar to that of silkworm in its tertiary structure (**Figure 3A**). Multiple sequence alignment analysis revealed that ApDNMT-1 has the highest similarity to *B. mori* (77.96%), followed by *Athalia rosae* (52.12%), *Ceratina calcarata* (49.74%), and so on. The full-length sequence of ApDNMT-2 contains an 1833 bp nucleotide sequence. The ORF of ApDNMT-2 was 888bp in length, with a 5′ UTR of 205 bp and a 3′ UTR of 740 bp. The deduced ApDNMT-2 protein contains 295 amino acid residues. The predicted molecular weight and isoelectric point are 33.89 kDa and 6.01, respectively. In a domain analysis of ApDNMT-2, we observed that this protein consisted of only one domain (the typical DNMT-2 cyt_C5_DNA methylase superfamily domain of DNMT-2 proteins), and its tertiary structure was highly comparable to that of the *B. mori* DNMT-2 protein (**Supplementary Figure S2** and **Figures 2**, **3B**). Additionally, multiple sequence alignment analysis revealed that ApDNMT-2 shares the maximum similarity with *B. mori* (66.78%), followed by *Tribolium castaneum* (48.41%), *Drosophila melanogaster* (43.73%), and other species. A phylogenetic analysis of amino acid sequences from diverse invertebrate and vertebrate species was carried out in order to better understand the evolutionary relationships of ApDNMT-1 and ApDNMT-2. The phylogenetic tree was constructed using the neighbor-joining method, which was performed using the Mega 6 software. The results revealed that ApDNMT-1 and ApDNMT-2 proteins are associated with the DNMT-1 and DNMT-2 proteins of animals, respectively (**Figure 4**).

### Spatial Expression Patterns of *ApDNMT-1* and *ApDNMT-2*

**Figure 5:**
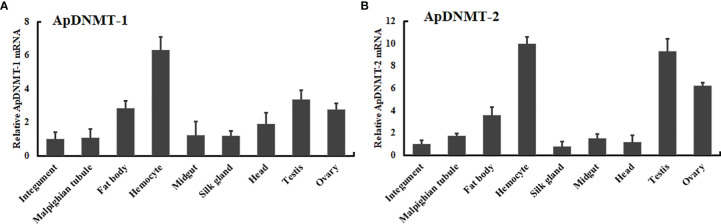
Tissue distribution profiles of *ApDNMT-1* and *ApDNMT-2* in various tissues of *A. pernyi* **(A)** Analysis of *ApDNMT-1* transcript expression in various tissues of silkworm larvae by qRT-PCR, **(B)** the *ApDNMT-2* transcript expression in various tissues of silkworm larvae by qRT-PCR. The mRNA level in the midgut was used as the calibrator. Bars exhibit the mean ± S.E (n = 3).

To understand the biological role of *ApDNMT-1* and *ApDNMAT-2*, we measured their transcription levels in different tissues (hemocyte, fat body, midgut, Malpighian tubules, integument, head, testis, ovary, and silk gland) of *A. pernyi* by performing qRT-PCR analysis. Using a tissue-specific mRNA expression analysis, it was found that ApDNMT-1 was transcribed in all of the tested tissues, with mRNA expression levels being notably high in the ovary, hemocytes, and fat bodies (**Figure 5A**). The *ApDNMT-2* gene was observed in all of the tissues examined, although its mRNA expression levels varied in each tissue. *ApDNMT-2* expression levels were shown to be higher in the ovary, hemocytes, and fat bodies, similar to ApDNMT-1 expression levels (**Figure 5B**). These findings show that these proteins have a broader range of biological roles in insects.

### *A. pernyi* DNMTs Induction Profile Analysis

**Figure 6:**
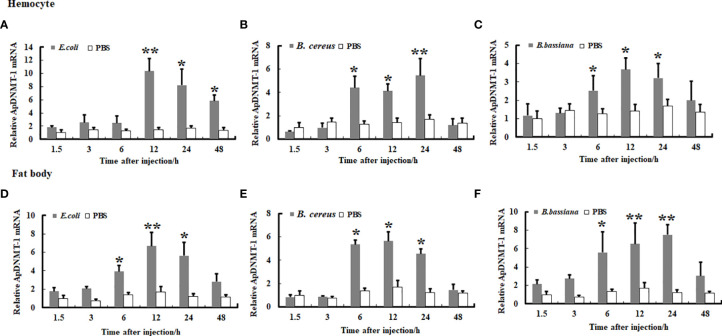
Expression profile of *ApDNMT-1* in hemocyte and fat body following microbial challenge. **(A)** Expression profile following *E. coli* challenge in hemocyte, **(B)** Expression profile following *B. cereus* challenge in hemocyte, **(C)** Expression profile following *B. bassiana* challenge in hemocyte, **(D)** Expression profile following *E. coli* challenge in the fat body, **(E)** Expression profile following *B*. *cereus* challenge in the fat body, **(F)** Expression profile following *B. bassiana* challenge in the fat body. PBS was injected as the control. Bars show mean ± S.E. (n = 3), and asterisks indicate significant differences (*p < 0.05, **p < 0.01).

**Figure 7:**
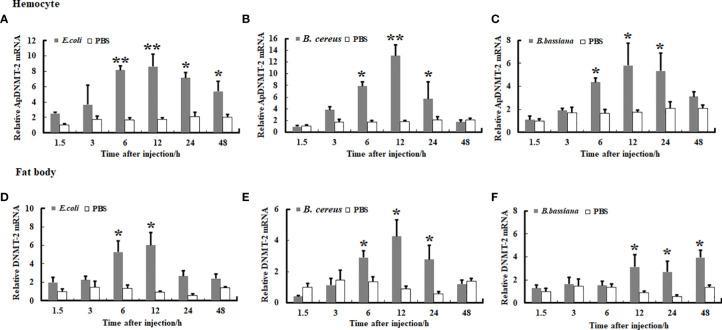
Expression profile of *ApDNMT-2* in hemocyte and fat body following microbial challenge. **(A)** Expression profile following *E. coli* challenge in hemocyte, **(B)** Expression profile following *B. cereus* challenge in hemocyte, **(C)** Expression profile following *B. bassiana* challenge in hemocyte, **(D)** Expression profile following *E. coli* challenge in the fat body, **(E)** Expression profile following *B. cereus* challenge in fat body, **(F)** Expression profile following *B. bassiana* challenge in the fat body. PBS was injected as the control. Bars show mean ± S.E. (n = 3), and asterisks indicate significant differences (*p < 0.05, **p < 0.01).

We examined the transcription levels of the *ApDNMT-1* and *ApDNMT-2* genes in hemocytes and fat bodies from *A. pernyi* at 1.5, 3, 6, 12, 24, and 48 h after injection with *B. bassiana, E. coli*, and *B. cereu* to investigate the influence of fungal and bacterial infections on *ApDNMT-1* and *ApDNMT-2* genes. The qRT-PCR analysis revealed that both fungal and bacterial pathogens induced transcription patterns of the *ApDNMT-1* and *ApDNMT-2* genes in hemocytes and fat bodies that were similar to one another. During the early hours after infection, the expression levels of *ApDNMT-1* and *ApDNMT-2* genes were slightly altered. *ApDNMT-1* gene expression was shown to be notably upregulated in the hemocytes and fat bodies at critical timepoints of infection (e.g., 6, 12, 24 h) (**Figure 6**). After the fungal and bacterial infections, the transcription patterns of ApDNMT-2 were found to be approximately similar to those of ApDNMT-1 (**Figure 7**). Following these timepoints, however, the transcription levels of these genes start to drop.

### Inhibition of DNMTs by 5-AZA Affected the Bacterial Replication and Mortality of *A. pernyi*

**Figure 8:**
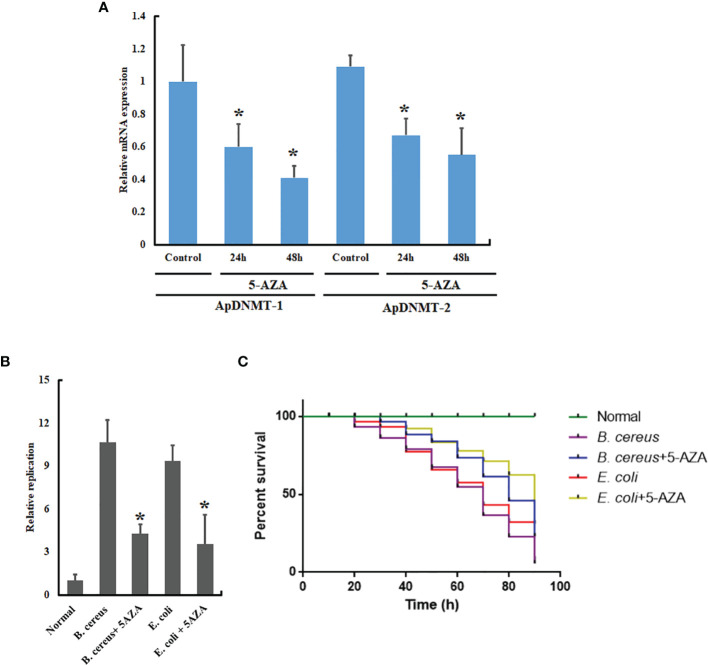
5-AZA administration reduced replication rates of the pathogenic bacteria and mortality rate of *A*. *pernyi.* **(A)** Relative mRNA expression levels of ApDNMT-1 and ApDNMT-2 in the 5-AZA-treated larvae and control group after 24 h and 48 h by qRT PCR. **(B)** qRT-PCR replication rate analysis showed that treatment of *A*. *pernyi* larvae with 5-AZA reduced replication rates of *B*. *cereus* and *E. coli* in *A*. *pernyi*. **(C)** 5-AZA application also decreased the mortality rate of *A. pernyi* following infection with the pathogenic bacteria. Bacterial DNA was quantified with universal 16S rRNA primers, and insect 18S rRNA was used as the reference control. Bars show mean ± S.E. (n = 3), and asterisks indicate significant differences (*p < 0.05).

Because of the remarkable variation in *ApDNMT-1* and *ApDNMT-2* gene transcription that was observed in *A. pernyi* larvae after fungal and bacterial infections, we next planned to investigate the importance of *ApDNMT-1* and *ApDNMT-2* involvement in innate immune responses. For this reason, we inhibited the DNMTs in the fifth instar larvae by injecting 5-AZA into the larvae, followed by *B. cereus* and *E. coli* injections. Before bacterial injection, we confirmed the suppression of *ApDNMT-1* and *ApDNMT-2* following the 5-AZA administration (**Figure 8A**). Total genomic DNA was extracted from the hemolymph samples of treated (bacterial-injected and 5-AZA treated) and control larvae. To perform qRT-PCR, we used 500 ng total genomic DNA for each qRT-PCR reaction, and the DNA concentrations were measured using a spectrophotometer. In qRT-PCR, specific primers for the 16s rRNA of the bacteria were used, and the insect 18s rRNA gene was used as an internal control for normalization. For each experiment, three biological replicates were replicated three times. Our results showed that 5-AZA treatment of larvae reduced replication of the pathogenic bacteria when compared to control larvae (**Figures 8A, B**). We also analyzed the mortality rate of bacteria-infected larvae and found that injection of 5-AZA attenuated larval mortality (**Figure 8C**).

### Antimicrobial Activity of the 5-AZA Treated Plasma

**Figure 9:**
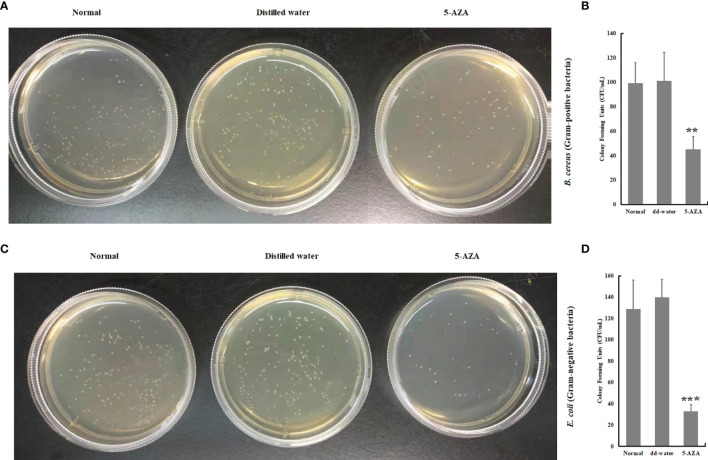
The plasma anti-bacterial activity was increased in the 5-AZA, and gram-positive bacteria **(A, B)** or 5-AZA and gram-negative bacteria **(C, D)** treated larval hemolymph. Plasma was sampled from *A. pernyi* treated with 5-AZA (n = 3) and distilled water. Equal volumes (10 μl) of plasma and bacterial suspension were incubated at 25°C for 1 h. Asterisks indicate significant differences (**p < 0.01, ***p < 0.001).

In order to determine whether the presence of 5-AZA, a DNA methylation inhibitor, modulates the antimicrobial activity, we carried out a bacterial clearance assay using plasma collected from larvae that had been treated with 5-AZA and bacteria. The results revealed that plasma from *A. pernyi* larvae treated with 5-AZA had significantly greater antibacterial activity against gram-positive and gram-negative bacteria than in control groups. For example, the survival rates of bacteria (*B. cereus* and *E. coli*) in the 5-AZA treated larvae were much lower than in the distilled water treated larvae and the untreated control larvae (**Figure 9**).

### Effects of DNMTs Inhibition on AMPs and Their Associated Pathways

**Figure 10:**
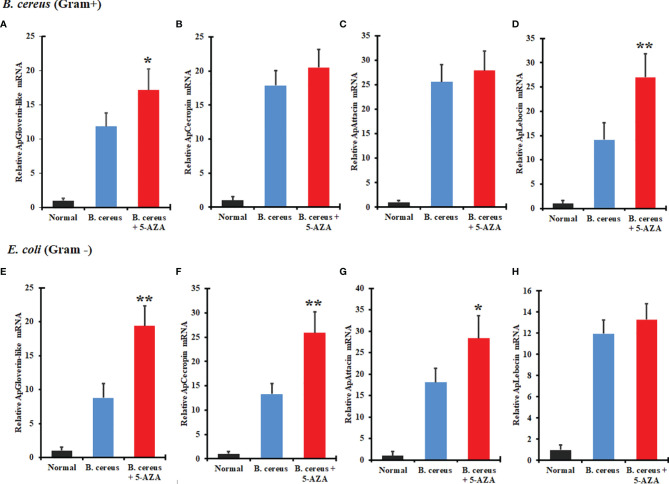
Treatment with 5-AZA modulated the expression of antimicrobial peptides (AMPs) in *A. pernyi* following bacterial infections. RT-qPCR analysis of gloverin-like **(A, E)**, cecropin **(B, F)**, attacin **(C, G)**, and lebocin **(D, H)** confirmed that 5-AZA administration altered the expression levels of AMPs in response to *B cereus* **(A–D)** and *E. coli* **(E–H)**. 18S was used as the reference gene in RT-qPCR. Reactions from three biological replicates were repeated three times. Bars show mean ± S.E. (n = 3), and asterisks indicate significant differences (*p < 0.05, **p < 0.01).

**Figure 11:**
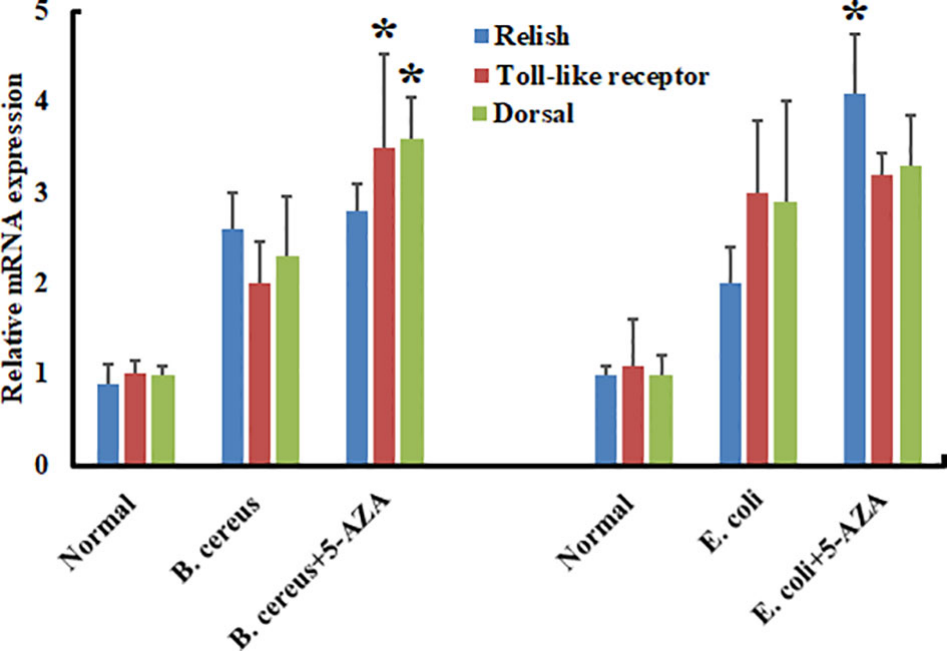
Treatment with 5-AZA modulated the expression of Toll and IMP pathway-associated genes in *A. pernyi* following bacterial infections. RT-qPCR analysis of relish, Toll-like receptor, and dorsal confirmed that 5-AZA administration altered the expression levels of these pathways-associated genes in response to *B cereus* and *E. coli*. 18S was used as the reference gene in RT-qPCR. Reactions from three biological replicates were repeated three times. Bars show mean ± S.E. (n = 3), and asterisks indicate significant differences (*p < 0.05).

To evaluate the effects of 5-AZA on AMP gene expression, we quantified changes in the transcript levels of various AMPs in larvae that had been treated with 5-AZA and then challenged with bacteria. We injected *A. pernyi* larvae in the control group with distilled water or with bacteria only. We observed that among the four AMPs analyzed, the expression of attacin, cecropin with Gram-positive and gloverin-like, attacin, and lebocin with gram-positive bacteria was strongly increased in 5-AZA-injected larvae after challenge with *B. cereus* and *E. coli* as compared to the control group (**Figure 10**). To understand further how 5-AZA and bacterial treatments affect these AMP-associated pathways, we analyzed the Toll and IMD pathway-associated genes (Toll-like receptor, dorsal, and relish), and the results revealed that the expression of these genes was altered in the 5-AZA and bacterial treated larvae compared to control groups (**Figure 11**).

## Discussions

In the present study, we identified two members of the DNA methyltransferase family, including DNMT-1 and DNMT-2, from the Chinese oak silkworm *A. pernyi*. These genes share high similarities with the DNMT-1 and DNMT-2 of other lepidopterans, particularly with *B. mori*. Previous studies have also reported the presence of only DNMT-1 and DNMT-2 genes in lepidopteran species (21, 28). These studies support the hypothesis of the loss of the DNMT-3 gene from lepidopteran species (29). Conserved domain analysis showed that ApDNMT-1 and ApDNMT-2 proteins have the typical domain composition of DNMT families. ApDNMT-1 has four conserved domains, while ApDNMT-2 has only 1 domain, suggesting that these proteins have different functions. The phylogenetic analysis of DNMTs also exhibited high similarities of ApDNMT-1 and ApDNMT-2 proteins to the homologous proteins in other lepidopterans, particularly *B. mori*. In the phylogenetic tree, we observed that DNMT-1 and DNMT-2 clades clustered separately, further strengthening the idea that these proteins have different biological roles in insects.

Furthermore, we analyzed the tissue distribution of these genes in *A. pernyi* larvae. Our tissue mRNA expression analysis showed that *ApDNMT-1* and *ApDNMT-2* expression was detectable in all *A. pernyi* larval tissues examined, but there were notable mRNA expression levels in the hemocytes, testis, and fat bodies. We presume that these variations in *ApDNMT-1* and *ApDNMT-2* transcription levels between tissues might have to do with different functions in insects. We suggest this possibility since many previous reports on insects have shown that DNA methylation regulates various physiological processes by modulating a wide range of gene expressions (21, 30). Kausar et al. (21) reviewed that in the insect’s DNA methylation levels of embryos, larvae, and adults, genes associated with innate immunity, development, growth, etc., respond to the changing levels of DNA methylation. In a previous study, the authors suggested constitutive expression of DNMTs in the insect tissues through their life cycle. This observation might be elucidated by the fact that insects undergo various physical and physiological changes during the life cycle, requiring a precise mechanism for the surveillance of these processes (21, 30). It is likely that we are seeing a similar factor affecting the expression of DNMTs. Because of the reasons above, it is not surprising that DNMTs are expressed constitutively in various tissues of *A. pernyi* larvae.

Moreover, mRNA expression analysis revealed that both ApDNMT-1 and ApDNMT-2 gene transcription levels were increased in the Chinese oak silkworm, *A. pernyi*, after microbial injections, particularly at critical timepoints of infection. The upregulation of DNMT genes transcription seems to be a general response of insects against microbial pathogens. Baradaran and his coworkers (30) reported the induction of DNMT-1 and DNMT-2 genes following bacterial infection. Besides, it has also been shown that *E. coli* infection induced DNMT-1 and DNMT-2 transcription levels and caused differential DNA methylation status of the genome in *Manduca sexta* (31) and *Galleria mellonella* (32). In addition, in *Aedes aegypti* and *Drosophila melanogaster*, increased DNMT-2 gene expression and hypermethylation have been observed following the Wolbachia infection (33–35). Although DNMT-3, a *de novo* DNA methyltransferase, is absent in lepidopteran insects, including *A. pernyi*, recent research has shown that in insects, particularly lepidopteran insects, DNMT-1 is responsible for DNA methylation rather than the DNMT-3 gene (28). In addition, this gene has also been reported to be involved in the maintenance of DNA methylation and also in *de novo* DNA methylation (36). In contrast, the presence of DNMT-2 in dipteran insects (e.g., *A. aegypti* and *D. melanogaster*) is responsible for changes in genome methylation (35, 37). The molecular mechanism by which pathogens modify DNA methylation is not clear in insects; however, this mechanism has been well explored in vertebrates (21). A previous study suggested that modulation of DNA methylation is caused by natural killer cell activity *via* IFN (interferon)-gamma (38). The superfamily of tripartite motif-containing proteins induces innate immunity and pathogen defense against a variety of pathogen infections in response to IFNs (39). Lim et al. found that the HBx-mediated reduction of TRIM22 may evade the antiviral effect of IFNs in a host, implying that HBx is one of the factors controlled by the pathogens to regulate DNA methylation. Subsequently, reducing IFN regulatory factor-1 binding affinity thereby decreases the IFN-stimulated induction of TRIM22h, which impedes IFN regulatory factor-1, which subsequently seems to influence DNA methyltransferase tool kit and DNA methylation (40). This process may help pathogens evade host immunity. Given our observations and these studies, it seems that the upregulation of ApDNMT-1 and ApDNMT-2 genes in *A. pernyi* after microbial infections can affect the DNA methylation status in this insect species, thereby modulating gene expression and affecting different physiological responses, including immunity.

Based on the above observations, we predict DNMT genes can affect the immune responses of the insect. Data are limited on the immunological functions of DNA methylation in insects, and we were interested in seeing whether DNA methylation might be implemented in the modulation of innate immune response in *A. pernyi*. For this purpose, we administrated 5-AZA to inhibit the activity of DNMT genes in *A. pernyi*. This DNMT inhibitor is commonly used to suppress DNA methylation in the genomes of insects (40–43). Our results revealed that bacterial replication and mortality rates were lower following 5-AZA treatment compared to the control group. Additionally, colony-forming units were also lower in the 5-AZA treated larvae, indicating that the DNMTs are essential for the optimal growth of bacteria in insects. To the best of our knowledge, there is only one report on the necessity of DNMTs for bacterial replication in insects (30). However, in humans, the tick-born bacterial pathogen *Anaplasma phagocytophilu*m greatly triggers hypermethylation in the neutrophil genome after 24 h post-infection, followed by differential expression of genes. In addition, the number of bacteria (*A. phagocytophilum*) growing in the presence of 5-AZA was approximately 25% less than in the control group (44).

Finally, we analyzed whether the mRNA expression of *A. pernyi* AMPs could be stimulated after an *E. coli* challenge and whether DNA methylation inhibition by 5-AZA had any effect on the transcription levels of these antimicrobial peptides. We observed that inhibition of DNMT inhibitor regulated AMPs following the bacterial infection. These results show that DNA methylation has a potential effect on the expression patterns of these peptides under bacterial infection, leading us to suggest that in *A. pernyi*, DNA methylation influences transcription of AMPs in response to Gram-negative *E. coli*. In humoral immunity, AMPs have a crucial role in suppressing microbial infection in insects (45, 46). Thus, the variation in the transcription levels of AMPs can shape the insect-bacterial pathogen interactions. Considering the increase of some AMPs transcription and the reduced colony-forming units and bacterial replication in *A. pernyi* following 5-AZA treatment, it also suggests that changes in the production of AMPs are one of the key molecular mechanisms that attenuate the replication of bacteria. In human cells, it has been shown that 5-AZA has an immunomodulatory function (47). In addition to insects, it has also been demonstrated in human that DNA methylation can be changed in a short period of time in response to microbial infection and that these changes in DNA methylation can influence immune cell responsiveness (48), suggesting that microbes, particularly bacteria, use DNA methylation strategies to evade host immune responses (49–51). A recent study analyzed the global transcriptional patterns of gene expression after *M. tuberculosis*, and found that the majority of differentially expressed genes were hypermethylated, which could play a biological role in the activation of innate and adaptive immune cells (51). *Helicobacter (H.) pylori* are also one of the most studied pathogens in terms of DNA methylation in humans. *H. pylori* causes changes in DNA methylation, and this abnormal DNA methylation in *H. pylori*-infected gastric mucosae has been linked to a higher risk of gastric cancer (52). DNA hypermethylation caused by *H. pylori* infection can be partially reversible once the bacterium is eliminated or 5-AZA is administered, resulting in a lower risk of gastric cancers triggered by *H. pylori* infection (53, 54). Besides, A non-catalytic biological role has also been described for the DNMT-1 gene since it can control expression levels without any variation in DNA methylation patterns, probably *via* interactions with other enzymatic proteins associated with epigenetic gene modulation (55, 56). Overall, whether the changes in the AMPs transcription patterns are due to the methylation status of these genes, or some other molecular mechanism, is still unexplored; however, the molecular mechanism could also be *via* variations in the gene transcriptional machinery triggered by the lack of DNMTs’ activity.

Furthermore, the upregulation of AMPs found in this study was mainly controlled by the Toll and IMD signaling pathways in insects, implying that DNA methylation may influence the activity of these pathways. Our analysis also confirmed that the 5-AZA and bacterial treatments influenced the expression of Toll and IMD pathway-associated genes. Our findings are consistent with previous studies; for example, evidence indicates that despite changes in AMPs transcription caused by DNA methylation (57–59), the Toll and IMD pathway-associated genes are modulated by epigenetic mechanisms, particularly by DNA methylation (60, 61). Thus, we conclude that DNA methylation influences the expression patterns of AMPs directly or indirectly, which helps a host organism to attenuate microbial infections.

In summary, our study is the first to identify and clone DNA methyltransferases (DNMT-1 and DNMT-2 genes) from the Chinese oak silkworm *A. pernyi*. Phylogenetic and structural analysis revealed a high similarity of these proteins with their respective homologous enzymes in other insects. ApDNMT-1 and ApDNMT-2 were observed in all tissues examined, and their transcription patterns changed in response to microbial infection. As a DNMTs inhibitor, 5-AZA treatment reduced the replication rate, colony-forming units, and mortality of larvae compared to the control group. Additionally, after 5-AZA treatment, transcription patterns of AMPs and their associated pathway-related genes were altered in response to bacterial infection. Altogether, our data uncovered a novel immune regulatory function of DNMTs that shapes the insect-pathogen interaction in the Chinese oak silkworm *A. pernyi*.

## Data Availability Statement

The original contributions presented in the study are included in the article/**Supplementary Material**. Further inquiries can be directed to the corresponding authors.

## Author Contributions

SK, MA, and IG: study design, data acquisition, results analysis, and prepared manuscript. RL, EZ, and QL: analysis of results and modification of the manuscript. HC and ML critical review, modification of manuscript and fund acquisition. All authors contributed to the article and approved the submitted version.

## Funding

This work was supported by the China Postdoctoral Science Foundation (2021M692676), Chongqing -Postdoctoral Science Foundation (cstc2021jcyj-bsh0128), the National Natural Science Foundation of China (31802142), the Doctorial Start-up Fund of Southwest University (SWU020023), the Fundamental Research Funds for the Central Universities (XDJK2019C089), and the Foundation of State Key Laboratory of Silkworm Genome Biology (SKLSGB-ORP202003).

## Conflict of Interest

The authors declare that the research was conducted in the absence of any commercial or financial relationships that could be construed as a potential conflict of interest.

## Publisher’s Note

All claims expressed in this article are solely those of the authors and do not necessarily represent those of their affiliated organizations, or those of the publisher, the editors and the reviewers. Any product that may be evaluated in this article, or claim that may be made by its manufacturer, is not guaranteed or endorsed by the publisher.

## Supplementary Material

Supplementary Figure 1
*ApDNMT-1* nucleotide and deduced amino acid sequence of *A. pernyi*. The *ApDNMT-1* deduced amino acid sequence is presented below the nucleotide sequence of cDNA, and the one-letter codes are aligned with the second nucleotide of each codon. The nucleotide and amino acid sequences are numbered at the left. Identified domains were labeled as DNMT1-RDF: DNA methyltransferase replication foci domain which is indicated by a red box, ZnF: Zinc finger domain is indicated by an orange colour box, BAH: Bromo adjacent homology domain is shown in yellow colour box, DCM: DNA-cytosine methyltransferases domain is shown in green box Cys-rich: cysteine-rich ADD domain is presented in a blue box.

Supplementary Figure 2
*ApDNMT-2* nucleotide and deduced amino acid sequence of *A. pernyi*. The *ApDNMT-2* deduced amino acid sequence is presented below the nucleotide sequence of cDNA, and the one-letter codes are aligned with the second nucleotide of each codon. The nucleotide and amino acid sequences are numbered at the left. The putative DNMT2 cyt_C5_DNA methylase superfamily domain is shown in the red box.

## References

[1] Li W Zhang Z Lin L Terenius O. *Antheraea pernyi* (Lepidoptera: Saturniidae) and Its Importance in Sericulture, Food Consumption, and Traditional Chinese Medicine. J Econ Entomol (2017) 110(4):1404–11. doi: 10.1093/jee/tox140 28535207

[2] Kausar S Abbas MN Qian C Zhu BJ Sun Y Sun YX. Serpin-14 Negatively Regulates Prophenoloxidase Activation and Expression of Antimicrobial Peptides in Chinese Oak Silkworm *Antheraea pernyi* . Dev Comp Immunol (2017) 76:45–55. doi: 10.1016/j.dci.2017.05.017 28545959

[3] Wang G Na S Qin L. Uncovering the Cellular and Humoral Immune Responses of *Antheraea pernyi* Hemolymph to *Antheraea pernyi* Nucleopolyhedrovirus Infection by Transcriptome Analysis. J Invert Pathol (2019) 166:107205. doi: 10.1016/j.jip.2019.107205 31136740

[4] Kausar S Abbas MN Qian C Zhu BJ Gao J Sun Y. Role of *Antheraea pernyi* Serpin 12 in Prophenoloxidase Activation and Immune Responses. Arch Insect Biochem Physiol (2018) 97:e21435. doi: 10.1002/arch.21435 29193264

[5] Hillyer JF. Insect Immunology and Hematopoiesis. Dev Comp Immunol (2016) 58:102–18. doi: 10.1016/j.dci.2015.12.006 PMC4775421 26695127

[6] Kausar S Abbas MN Zhao Y Cui H. Immune Strategies of Silkworm, *Bombyx mori* Against Microbial Infections. Invert Surviv J (2019) 16:130–40. doi: 10.25431/1824-307X/isj.v0i0.130-140

[7] Bestor TH. The DNA Methyltransferases of Mammals. Hum Mol Genet (2000) 9:2395–402. doi: 10.1093/hmg/9.16.2395 11005794

[8] Li E. Chromatin Modification and Epigenetic Reprogramming in Mammalian Development. Nat Rev Genet (2002) 3:662–73. doi: 10.1038/nrg887 12209141

[9] Xiang H Zhu J Chen Q Dai F Li X Li M. Single Base-Resolution Methylome of the Silkworm Reveals a Sparse Epigenomic Map. Nat Biotechnol (2010) 28:516–20. doi: 10.1038/nbt.1626 20436463

[10] Feliciello I Parazajder J Akrap I Ugarkovic D. First Evidence of DNA Methylation in Insect *Tribolium Castaneum*: Environmental Regulation of DNA Methylation Within Heterochromatin. Epigenetics (2013) 8:534–41. doi: 10.4161/epi.24507 PMC3741223 23644818

[11] Beeler SM Wong GT Zheng JM Bush EC Remnant EJ Oldroyd BP. Whole-Genome DNA Methylation Profile of the Jewel Wasp (*Nasonia Vitripennis*). G3 (Bethesda) (2014) 4:383–8. doi: 10.1534/g3.113.008953 PMC3962478 24381191

[12] Capuano F Mulleder M Kok R Blom HJ Ralser M. Cytosine DNA Methylation is Found in Drosophila Melanogaster But Absent in *Saccharomyces Cerevisiae*, *Schizosaccharomyces Pombe*, and Other Yeast Species. Anal Chem (2014) 86:3697–702. doi: 10.1021/ac500447w PMC4006885 24640988

[13] Glastad KM Hunt BG Yi SV Goodisman MA. DNA Methylation in Insects: on the Brink of the Epigenomic Era. Insect Mol Biol (2011) 20:553–65. doi: 10.1111/j.1365-2583.2011.01092.x 21699596

[14] Standage DS Berens AJ Glastad KM Severin AJ Brendel VP Toth AL. Genome, Transcriptome and Methylome Sequencing of a Primitively Eusocial Wasp Reveal a Greatly Reduced DNA Methylation System in a Social Insect. Mol Ecol (2016) 25:1769–84. doi: 10.1111/mec.13578 26859767

[15] Wu P Jie W Shang Q Annan E Jiang X Hou C. DNA Methylation in Silkworm Genome may Provide Insights Into Epigenetic Regulation of Response to *Bombyx mori* Cypovirus Infection. Sci Rep (2017) 7:16013. doi: 10.1038/s41598-017-16357-7 29167521 PMC5700172

[16] Lyko F Maleszka R. Insects as Innovative Models for Functional Studies of DNA Methylation. Trends Genet (2011) 27:127–31. doi: 10.1016/j.tig.2011.01.003 21288591

[17] Bonasio R Li Q Lian J Mutti NS Jin L Zhao H. Genome-Wide and Caste-Specific DNA Methylomes of the Ants Camponotus Floridanus and Harpegnathos Saltator. Curr Biol (2012) 22:1755–64. doi: 10.1016/j.cub.2012.07.042 PMC3498763 22885060

[18] Yan H Bonasio R Simola DF Liebig J Berger SL Reinberg D. DNA Methylation in Social Insects: How Epigenetics can Control Behavior and Longevity. Annu Rev Entomol (2015) 60:435–52. doi: 10.1146/annurev-ento-010814-020803 25341091

[19] Biergans SD Giovanni GC Judith R Charles C. Dnmts and Tettarget Memory-Associated Genes After Appetitive Olfactory Training in Honey Bees. Sci Rep (2015) 5:21656. doi: 10.1038/srep16223 PMC4632027 26531238

[20] Herb BR Wolschin F Hansen KD Aryee MJ Langmead B Irizarry R. Reversible Switching Between Epigenetic States in Honeybee Behavioral Subcastes. Nat Neurosci (2012) 15:1371–3. doi: 10.1038/nn.3218 PMC3518384 22983211

[21] Kausar S Abbas MN Cui H. A Review on the DNA Methyltransferase Family of Insects: Aspect and Prospects. Int J Biol Macromol (2021) 186:289–302. doi: 10.1016/j.ijbiomac.2021.06.205 34237376

[22] Ye YH Woolfit M Huttley GA Rances E Caragata EP Popovici J. Infection With a Virulent Strain of Wolbachia Disrupts Genome Wide-Patterns of Cytosine Methylation in the Mosquito *Aedes Aegypti* . PloS One (2013) 8:e66482. doi: 10.1371/journal.pone.0066482 23840485 PMC3686743

[23] Vilcinkas A. The Role of Epigenetics in Host–Parasite Coevolution: Lessons From the Model Host Insects *Galleria Mellonella* and *Tribolium Castaneum* . Zoology (2016) 119:273–80. doi: 10.1016/j.zool.2016.05.004 27341739

[24] Bacchetti De Gregoris T Aldred N Clare AS Burgess JG. Improvement of Phylum- and Class-Specific Primers for Real-Time PCR Quantification of Bacterial Taxa. J Microbiol Methods (2011) 86:351–6. doi: 10.1016/j.mimet.2011.06.010 21704084

[25] Livak KJ Schmittgen TD. Analysis of Relative Gene Expression Data Using Real-Time Quantitative PCR and the 2(-Delta Delta CT) Method. Methods (2001) 25:402–8. doi: 10.1006/meth.2001.1262 11846609

[26] Christman JK. 5-Azacytidine and 5-aza-2′-deoxycytidine as Inhibitors of DNA Methylation: Mechanistic Studies and Their Implications for Cancer Therapy. Oncogene (2002) 21:5483–95. doi: 10.1038/sj.onc.1205699 12154409

[27] Abbas MN Kausar S Gul I Ke XX Dong Z Lu X. Suppressor of Cytokine Signalling 6 is a Potential Regulator of Antimicrobial Peptides in the Chinese Oak Silkworm, Antheraea pernyi. Mole Immunol (2021) 140:12–21. doi: 10.1016/j.molimm.2021.10.001 34628136

[28] Bewick AJ Vogel KJ Moore AJ Schmitz RJ. Evolution of DNA Methylation Across Insects. Mol Biol Evol (2017) 34:654–65. doi: 10.1093/molbev/msw264 PMC5400375 28025279

[29] Misof B Liu S Meusemann K Peters RS Donath A Mayer C. Phylogenomics Resolves the Timing and Pattern of Insect Evolution. Science (2014) 346:763–7. doi: 10.1126/science.1257570 25378627

[30] Baradaran E Moharramipour S Asgari S Mehrabadi M. Induction of DNA Methyltransferase Genes in *Helicoverpa Armigera* Following Injection of Pathogenic Bacteria Modulates Expression of Antimicrobial Peptides and Affects Bacterial Proliferation. J Insect Physiol (2019) 118:103939. doi: 10.1016/j.jinsphys.2019.103939 31493391

[31] Heitmueller M Billion A Dobrindt U Vilcinskas A Mukherjee K. Epigenetic Mechanisms Regulate Innate Immunity Against Uropathogenic and Commensal-Like *Escherichia Coli* in the Surrogate Insect Model *Galleria Mellonella* . Infect Immun (2017) 85:e00336–00317. doi: 10.1128/IAI.00336-17 PMC5607417 28739824

[32] Gegner J Baudach A Mukherjee K Halitschke R Vogel H Vilcinskas A. Epigenetic Mechanisms are Involved in Sex-Specific Trans-Generational Immune Priming in the Lepidopteran Model Host *Manduca Sexta* . Front Physiol (2019) 10:137. doi: 10.3389/fphys.2019.00137 30886585 PMC6410660

[33] Zhang G Hussain M O’Neill SL Asgari S. Wolbachia Uses a Host microRNA to Regulate Transcripts of a Methyltransferase, Contributing to Dengue Virus Inhibition in *Aedes Aegypti* . Proc Natl Acad Sci USA (2013) 110:10276–81. doi: 10.1073/pnas.1303603110 PMC3690878 23733960

[34] LePage DP Jernigan KK Bordenstein SR. The Relative Importance of DNA Methylation and Dnmt2-mediated Epigenetic Regulation on Wolbachia Densities and Cytoplasmic Incompatibility. PeerJ (2014) 2:e678. 25538866 10.7717/peerj.678 PMC4266856

[35] Bhattacharya T Newton ILG Hardy RW. Wolbachia Elevates Host Methyltransferase Expression to Block an RNA Virus Early During Infection. PloS Pathog (2017) 13:e1006427. doi: 10.1371/journal.ppat.1006427 28617844 PMC5472326

[36] Yarychkivska O Tavana O Gu W Bestor TH. Independent Functions of DNMT1 and USP7 at Replication Foci. Epigenet Chromatin (2018) 11:9. doi: 10.1186/s13072-018-0179-z PMC5828336 29482658

[37] Kunert N Marhold J Stanke J Stach D Lyko F. A Dnmt2-like Protein Mediates DNA Methylation in *Drosophila* . Development (2003) 130:5083–90. doi: 10.1242/dev.00716 12944428

[38] Okamoto Y Shinjo K Shimizu Y Sano T Yamao K Gao W. Hepatitis Virus Infection Affects DNA Methylation in Mice With Humanized Livers. Gastroenterology (2014) 146:562–72. doi: 10.1053/j.gastro.2013.10.056 24184133

[39] Ozato K Shin DM Chang TH Morse HC. TRIM Family Proteins and Their Emerging Roles in Innate Immunity. Nat Rev Immunol (2008) 8:849–60. doi: 10.1038/nri2413 PMC3433745 18836477

[40] Lim KH Park ES Kim DH Cho KC Kim KP Park YK. Suppression of Interferon-Mediated anti-HBV Response by Single CpG Methylation in the 5’-UTR of TRIM22. Gut (2018) 67:166–78. doi: 10.1136/gutjnl-2016-312742 28341749

[41] Amarasinghe HE Clayton CI Mallon EB. Methylation and Worker Reproduction in the Bumble-Bee (*Bombus Terrestris*). Proc R Soc Lond B Biol Sci (2014) 281:20132502. doi: 10.1098/rspb.2013.2502 PMC4027386 24523266

[42] Kumar S Kim Y. An Endoparasitoid Wasp Influences Host DNA Methylation. Sci Rep (2017) 7:43287. doi: 10.1038/srep43287 28230192 PMC5322367

[43] Xu G Zhang J Lyu H Song Q Feng Q Xiang H. DNA Methylation Mediates BmDeaf1-regulated Tissue-and Stage-Specific Expression of BmCHSA-2b in the Silkworm, *Bombyx mori* . Epigenet Chromatin (2018) 11:32. doi: 10.1186/s13072-018-0202-4 PMC6001065 29903048

[44] Sinclair SHG Yegnasubramanian S Dumler JS. Global DNA Methylation Changes and Differential Gene Expression in *Anaplasma Phagocytophilum*-Infected Human Neutrophils. Clin Epigenet (2015) 7:77. doi: 10.1186/s13148-015-0105-1 PMC4518890 26225157

[45] Abbas MN Zhu BJ Kausar S Dai LS Sun YX Tian JW. Suppressor of Cytokine Signaling 2-12 Regulates Antimicrobial Peptides and Ecdysteroid Signaling Pathways in *Bombyx mori* (Dazao). J Insect Physiol (2017) 103:47–56. doi: 10.1016/j.jinsphys.2017.10.004 29032156

[46] Zhang K Hu X Zhao Y Pan G Li C Ji H. Scavenger Receptor B8 Improves Survivability by Mediating Innate Immunity in Silkworm, *Bombyx mori* . Dev Comp Immunol (2021) 116:103917. doi: 10.1016/j.dci.2020.103917 33159959

[47] Sanchez-Abarca LI Gutierrez-Cosio S Santamaria C Caballero-Velazquez T Blanco B Herreo-Sanchez C. Immunomodulatory Effect of 5-Azacytidine (5-azaC): Potential Role in the Transplantation Setting. Blood (2010) 115:107–21. doi: 10.1182/blood-2009-03-210393 19887673

[48] Pacis A Tailleux L morin AM Lambourne J MacIsaac JL Yotova V. Bacterial Infection Remodels the DNA Methylation Landscape of Human Dendritic Cells. Genome Res (2015) 25(12):1801–11. doi: 10.1101/gr.192005.115 PMC4665002 26392366

[49] Das K Saikolappan S Dhandayuthapani S. Differential Expression of miRNAs by Macrophages Infected With Virulent and Avirulent Mycobacterium Tuberculosis. Tuberculosis (Edinb) (2013) 93 Suppl:S47–50. doi: 10.1016/S1472-9792(13)70010-6 24388649

[50] Das K Garnica O Dhandayuthapani S. Modulation of Host miRNAs by Intracellular Bacterial Pathogens. Front Cell Infect Microbiol (2016) 6:79. doi: 10.3389/fcimb.2016.00079 27536558 PMC4971075

[51] Looney M Lorenc R. Halushka MK and Karakousis Pc. Key Macrophage Responses to Infection With Mycobacterium Tuberculosis are Co-Regulated by microRNAs and DNA Methylation. Front Immunol (2021) 12: 685237. doi: 10.3389/fimmu.2021.685237 34140955 PMC8204050

[52] Maekita T Nakazawa K Mihara M Nakajima T Yanaoka K Iguchi M. High Levels of Aberrant DNA Methylation in *Helicobacter Pylori*-Infected Gastric Mucosae and its Possible Association With Gastric Cancer Risk. Clin Cancer Res (2006) 12:989–95. doi: 10.1158/1078-0432.CCR-05-2096 16467114

[53] Sepulveda AR Yao Y Yan W Park DI Kim JJ Gooding W. Cpg Methylation and Reduced Expression of O6-methylguanine DNA Methyltransferase is Associated With *Helicobacter Pylori* Infection. Gastroenterology (2010) 138:1836–44. doi: 10.1053/j.gastro.2009.12.042 20044995

[54] Niwa T Toyoda T Tsukamoto T mori A Tatematsu M Ushijima T. Prevention of *Helicobacter Pylori*-Induced Gastric Cancers in Gerbils by a DNA Demethylating Agent. Cancer Prev Res (Phila) (2013) 6:263–70. doi: 10.1158/1940-6207.CAPR-12-0369 23559452

[55] Clements N Hannigan M Miller SL Peel JL Milford JB. Comparisons of Urban and Rural PM10–2.5 and PM2.5 Mass Concentrations and Semi-Volatile Fractions in Northeastern Colorado. Atmos Chem Phys (2016) 16:7469–84. doi: 10.5194/acp-16-7469-2016

[56] Schulz JM Knoflach F Hernandez MC Bischofberger J. Dendrite-Targeting Interneurons Control Synaptic NMDA-Receptor Activation *Via* Nonlinear A5-GABAA Receptors. Nat Commun (2018) 9:3576. doi: 10.1038/s41467-018-06004-8 30177704 PMC6120902

[57] Noh YH Lee J Seo SJ. Myung SCh. Promoter DNA Methylation Contributes to Human β-Defensin-1 Deficiency in Atopic Dermatitis. Anim Cells Sys (2018) 22:172–7. doi: 10.1080/19768354.2018.1458652 PMC6138329 30460095

[58] Chen X Qi G Qin M Zou Y Zhong K Tang Y. DNA Methylation Directly Downregulates Human Cathelicidin Antimicrobial Peptide Gene (CAMP) Promoter Activity. Oncotarget (2017) 8:27943–52. doi: 10.18632/oncotarget.15847 PMC5438620 28427192

[59] Kweh MF Merriman KE Nelson CD. Inhibition of DNA Methyltransferase and Histone Deacetylase Increases β-Defensin Expression But Not the Effects of Lipopolysaccharide or 1,25-Dihydroxyvitamin D3 in Bovine Mammary Epithelial Cells. J Dairy Sci (2018) 102:5706–12. doi: 10.3168/jds.2018-16141 30954263

[60] Goto A Fukuyama H Imler JL Hoffmann JA. The Chromatin Regulator DMAP1 Modulates Activity of the Nuclear Factor _B (Nf-_B) Transcription Factor Relish in the Drosophila Innate Immune Response. J Biol Chem (2014) 289:20470–6. doi: 10.1074/jbc.C114.553719 PMC4110259 24947515

[61] Bind MA Zanobetti A Gasparrini A Peters A Coull B Baccarellia A. Effects of Temperature and Relative Humidity on DNA Methylation. Epidemiology (2014) 25:561–9. doi: 10.1097/EDE.0000000000000120 PMC4224120 24809956

