# Supplementary figures and images for: Molecular Identification of Two DNA Methyltransferase Genes and Their Functional Characterization in the Anti-Bacterial Immunity of Antheraea pernyi
Source: Front Immunol. 2022 May 16;13:855888. doi: 10.3389/fimmu.2022.855888 (PMC9149099; doi:10.3389/fimmu.2022.855888)

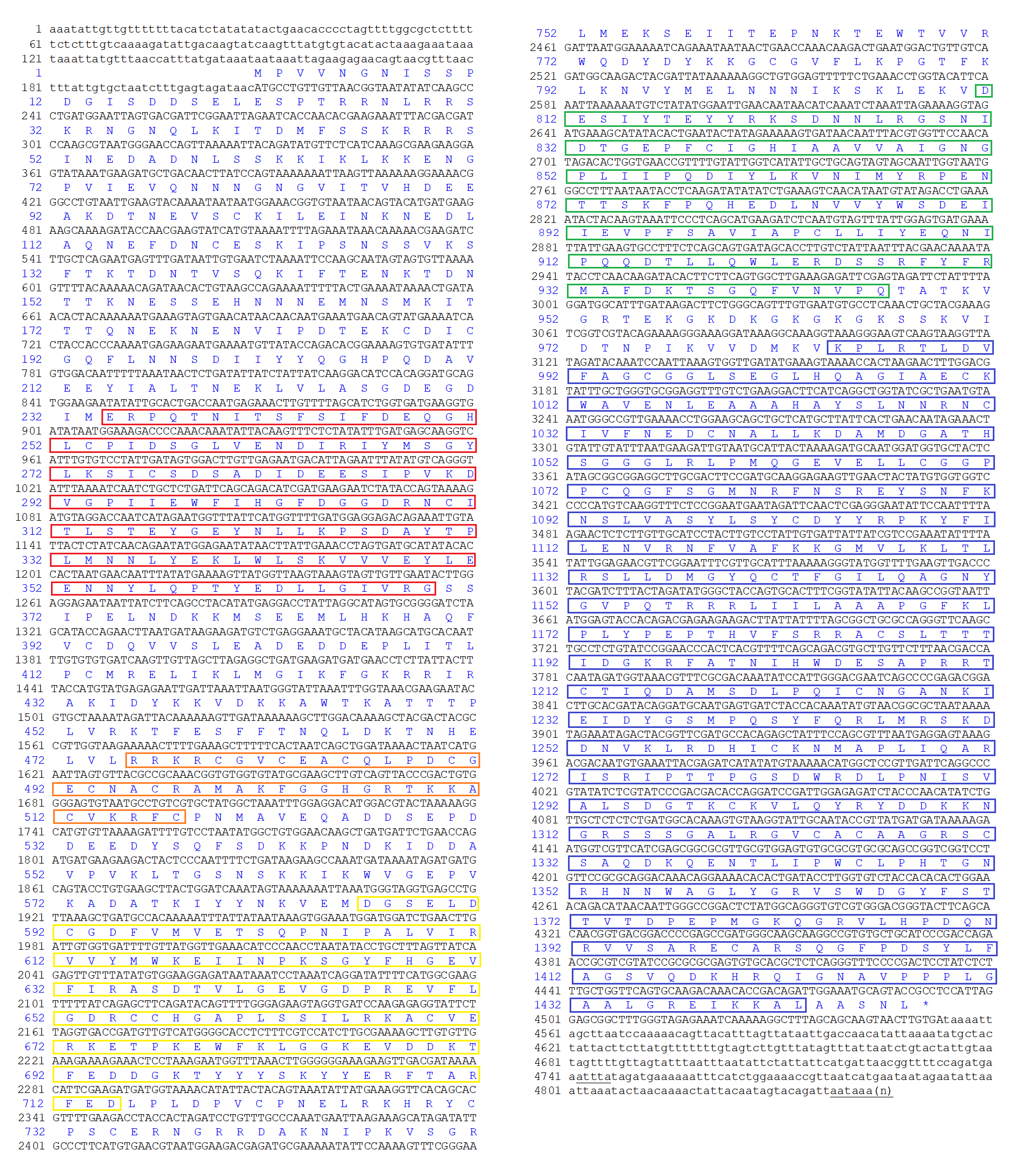

Supplement: Supplementary Figure 1 — ApDNMT-1 nucleotide and deduced amino acid sequence of A. pernyi. The ApDNMT-1 deduced amino acid sequence is presented below the nucleotide sequence of cDNA, and the one-letter codes are aligned with the second nucleotide of each codon. The nucleotide and amino acid sequences are numbered at the left. Identified domains were labeled as DNMT1-RDF: DNA methyltransferase replication foci domain which is indicated by a red box, ZnF: Zinc finger domain is indicated by an orange colour box, BAH: Bromo adjacent homology domain is shown in yellow colour box, DCM: DNA-cytosine methyltransferases domain is shown in green box Cys-rich: cysteine-rich ADD domain is presented in a blue box. [file Image_1.tif]

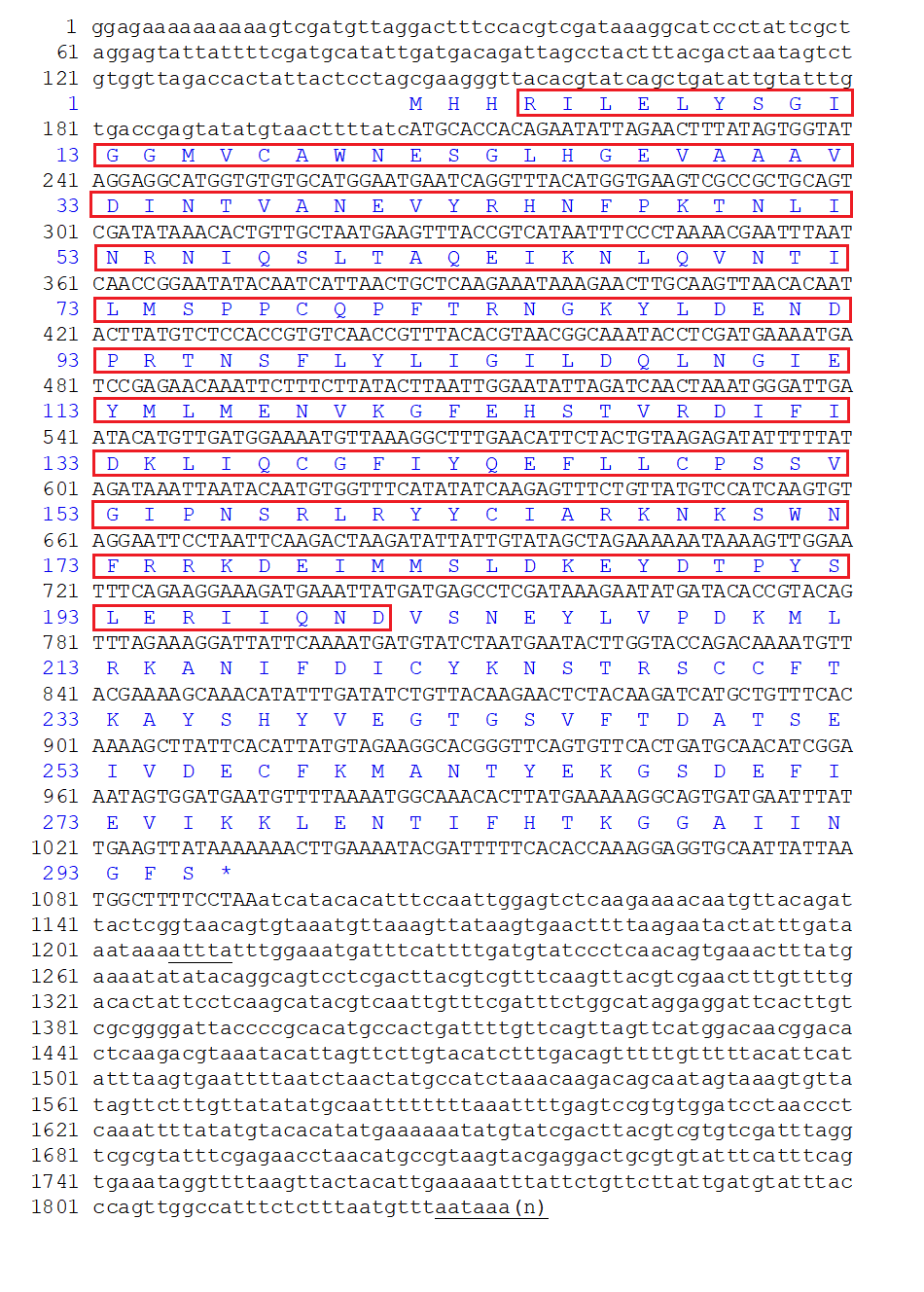

Supplement: Supplementary Figure 2 — ApDNMT-2 nucleotide and deduced amino acid sequence of A. pernyi. The ApDNMT-2 deduced amino acid sequence is presented below the nucleotide sequence of cDNA, and the one-letter codes are aligned with the second nucleotide of each codon. The nucleotide and amino acid sequences are numbered at the left. The putative DNMT2 cyt_C5_DNA methylase superfamily domain is shown in the red box. [file Image_2.tif]
